# Alterations in Administration of Maternity Benefit

**Published:** 1914-01-17

**Authors:** 


### NATIONAL INSURANCE ACT DEVELOPMENTS.

### Alterations in Administration of Maternity Benefit.

On Monday last, January 12, several important alterations affecting the administration of maternity benefit under the National Insurance Act came into operation. It is therefore necessary, in the interest of insured persons, to call attention to the new regulations, and to point out the way in which full advantage may be taken of them. The alterations that have now come into force carry into effect the provisions of the Amendment Act which was placed on the Statute Book last year. It may be noted, in passing, that section 43 of the Act of 1913 stipulates that the provisions of that Act should come into operation not later than January 15, 1914, so that the date fixed for the alteration in the administration of maternity is almost as late as the Insurance Commissioners had power to defer it.

There is no doubt that maternity benefit is one of the greatest boons conferred by the National Insurance scheme, and its authors did not fail to lay stress upon the advantages that were to accrue directly to the insured persons who would benefit by it, and indirectly to the community as a whole. Popular imagination was stirred by this benefit probably more than by any other, but it did not require many months of actual experience of administration to disclose certain fundamental errors both in the general principle upon which the benefit was granted and in the drafting of the Act. Thus it was that advantage was taken of the opportunity afforded by the Amendment Act to rectify as far as possible the hastily considered provisions of the original enactment where they had been found wanting. It is to be noted, however, that the provisions relating to the amendment of the maternity benefit did not find a place in the Bill as originally presented to Parliament. The proposals, which were eventually adopted in a slightly modified form, were brought forward in the Committee stage, and their consideration occupied more time than any other clauses of the Bill. Thus there are grounds for hoping that the amended rules will prove more satisfactory in operation than those of the original plan.

### Defects in the Original Scheme.

The first great defect that was revealed in the original provisions arose not through faulty drafting of clauses, or through the institution of complicated administrative machinery, but from the fact that inefficient safeguards were set up to provide that the benefit was not misused. The recipient under the principal Act was, in the majority of cases, the husband, whose receipt was the legal discharge for the payment to the approved society of which he was a member. It was only when the wife was an employed contributor, and the husband was not insured, that the benefit became absolutely the property of the wife.

The possible misuse of the money by the husband was to some extent foreseen, since section 19 of the principal Act provided that "where . . . maternity benefit is given or paid to the husband, it shall be the duty of the husband to make adequate provision to the best of his power for the maintenance and care of his wife during her confinement and for a period of four weeks after her delivery, and if he neglects or refuses to do so he shall be liable, upon summary conviction, to imprisonment, with or without hard labour, for any term not exceeding one month." This provision, however, was found to be of little use for the purpose for which it was designed, for the very good reason that the persons for whom the benefit is provided would not, as a general rule, care to seek the assistance of the Courts, except in case of absolute necessity. It was, moreover, shown by indisputable evidence that, in spite of the heavy penalty attaching to such an action, the maternity benefit, which was to have done so much for the mother in her hour of need, never reached her at all, having been squandered by the husband, in a sufficient number of cases to make it desirable to institute a change of procedure.

### The Mother's Benefit.

In recognition of this fact provision was made in the Amendment Act whereby maternity benefit, whether paid in respect of the wife's insurance or from that of the husband, is in all cases to be the property of the mother, and her receipt only, or that of her husband, if authorised by her, is to be a sufficient discharge to the approved society. In other words, the effect of the recent Act is to change maternity benefit from a benefit for the husband to a benefit for the mother.

It is this particular provision of the Act which came into operation on Monday last, and the change which it implies is more far-reaching than would be supposed by the casual observer. In order to emphasise the importance of the change an official circular has recently been sent to all approved societies by the Insurance Commissioners calling attention to the revised conditions that must apply before the benefit can be paid.

### Administrative Difficulties and a Solution.

The Insurance Commissioners are careful to point out that "when societies pay the benefit to the husband they must, for their own protection against a second claim from the wife, be satisfied that the wife has authorised the husband to receive the benefit, and they must realise that they are only completely protected against such a second claim (in cases where they do not pay to her and obtain her receipt) if they can produce a written authority from her to pay the husband."

It will at once be seen that there is here a considerable difficulty to be overcome. To be really, effective the maternity benefit must be paid promptly as soon as it becomes due. It cannot, of course, be paid until the approved society is furnished with evidence of the confinement. This will generally be easily obtained from the doctor or midwife in attendance. But there is also the necessity to obtain the mother's receipt or her written authority to pay her husband, and it will not always be possible for her to sign the necessary papers that will be required. This difficulty was raised in argument against the new provisions by the approved societies when the Bill was in Committee, but it should not prove to be insuperable. In most cases it will be possible for the wife to sign the form of receipt or the letter of authority for payment to be made to the husband some days in advance of the birth of the child, and in this way no difficulty need arise to delay the payment. It is, therefore, desirable that approved societies should furnish the forms of receipt for signature some time in advance of the expected event, and insured persons should take the trouble to see that the forms are completed. One of the principal causes for such dissatisfaction as has been expressed against the administration of the Act would have been avoided if the insured had taken more trouble to follow the requirements of their societies in such simple matters.

### Second Maternity Benefit.

The official circular, above referred to, also deals with another provision of the Amendment Act which came into force last Monday—namely, the provision of a second maternity benefit. It is enacted by section 14, sub-section (3) of the Act of 1913 that, instead of being entitled for the period of four weeks after confinement to any sickness or disablement benefit as under the principal Act, insured *married* women who are employed contributors, or, in the case of a posthumous child, insured widows, will be entitled to receive from their own society a second maternity benefit. It is important to notice that this provision only affects *married* women who are *employed contributors*. It does not affect unmarried women, who are expressly limited from the receipt of maternity benefit by the principal Act and debarred from sickness benefit. Married women are not entitled to become ordinary voluntary contributors, but they may become special voluntary contributors, paying at the reduced rate of 3d. a week. Such special voluntary contributors are not entitled to the double maternity benefit.

Furthermore, the second maternity benefit is to be paid by the woman's society, and is in lieu of the sickness or disablement benefit that would have been payable under the principal Act, with this difference, that the payment is not to count in reckoning the number of weeks' sick pay to which she is entitled should she remain, or subsequently become, incapable of work after the expiration of four weeks from the confinement.

The change in administration here indicated is important in two directions. In the first place it makes a total sum of £3—available in the particular circumstances at the time of, or very shortly after, the confinement, instead of spreading the payments of sickness benefit over the four weeks, if the incapacity for work lasted so long. Secondly, a very salutary condition has been imposed by legislation which makes it necessary that every approved society which has female members shall adopt a rule to the effect that the second maternity benefit can only be paid provided the woman abstains from remunerative work for a period of four weeks after her confinement. By this means it is hoped that both mother and infant will be cared for, and, although the mother is not prevented from doing her domestic duties when she is sufficiently recovered, she is debarred from that harder struggle for the means of livelihood which has so often been the cause of early breakdown and premature death of her offspring.

### A Point of Interest to Hospitals.

We have so frequently dealt with the effect of section 12 of the principal Act, relating to the payment by approved societies to hospitals, that it might be thought that the matter was exhausted. The provisions of the Amendment Act in regard to the second maternity benefit which are now in force have to be considered in reference to the subject. It is provided in the principal Act that if the inmate is a married woman or widow having dependants, and the sums payable include sums which would have been payable both on account of sickness or disablement benefit, *and*, on account of maternity benefit, no part of the latter benefit shall be paid or applied for the relief or maintenance of the dependants, but may be paid to the institution in a similar manner as if there were no dependants. This reservation of maternity benefit for the possible assistance of the institution in which the woman is confined is obviously just, and this reservation is not affected by the new arrangements. But this is the full extent of the allowance that may be made to the institution.

Although sickness benefit is no longer to be paid as such as well as maternity benefit, the second maternity benefit is distinguished from the first by the provision that it is not to be available as a grant towards the maintenance of the inmate while she is in the institution, but is to be applied for the benefit of her dependants. Thus the payment of the second maternity benefit to the hospital, or similar institution, is an impossibility. Knowing as we do what little advantage the hospitals have derived from the permissive powers conferred on the approved societies under section 12 of the principal Act it is perhaps as well that institutional managers should be aware that in this instance no benefit can be obtained and thus save themselves the trouble of seeking to make agreements with the approved societies.

There is one further provision of the Amendment Act which came into force on Monday last which should be noted—namely, that the maternity benefit payable in respect of the insurance of an alien whose wife before marriage was a British subject is to be increased in amount by two-sevenths. This is the Government contribution, and is to be provided out of moneys voted by Parliament. The effect of the alteration is to give the same benefit of 30s. to a woman on confinement when she has changed her nationality on marriage as if she had married a British subject.

